# Neurocognitive and Social Cognitive Approaches for Improving Functional Outcome in Early Psychosis: Theoretical Considerations and Current State of Evidence

**DOI:** 10.1155/2012/815315

**Published:** 2012-04-05

**Authors:** Cali F. Bartholomeusz, Kelly Allott

**Affiliations:** ^1^Melbourne Neuropsychiatry Centre, Department of Psychiatry, National Neuroscience Facility, The University of Melbourne, Level 2-3 Alan Gilbert Building, 161 Barry Street, Carlton South, VIC 3053, Australia; ^2^Orygen Youth Health Research Centre, Centre for Youth Mental Health, The University of Melbourne, Locked Bag 10, Parkville, VIC 3052, Australia

## Abstract

Improving functional outcome, in addition to alleviating psychotic symptoms, is now a major treatment objective in schizophrenia research. Given the large body of evidence suggesting pharmacological treatments generally have minimal effects on indices of functioning, research has turned to psychosocial rehabilitation programs. Among these, neurocognitive and social cognitive interventions are at the forefront of this field and are argued to target core deficits inherent to the schizophrenia illness. However, to date, research trials have primarily focused on chronic schizophrenia populations, neglecting the early psychosis groups who are often as severely impaired in social and occupational functioning. This theoretical paper will outline the rationale for investigating adjunctive cognitive-based interventions in the early phases of psychotic illness, critically examine the current approach strategies used in these interventions, and assess the evidence supporting certain training programs for improving functional outcome in early psychosis. Potential pathways for future research will be discussed.

## 1. Introduction

The field of schizophrenia research and treatment is in a transitional phase, shifting from a focus on outcomes that are measured by symptomatic remission and basic illness management to an all-encompassing outlook on real-world functional recovery [1, 2]. This is an exciting time for researchers and consumers, as the improvement in social, occupational, and personally challenging activities that impact the individual's day-to-day living are now primary research objectives. In this research era it is also widely acknowledged that the early phase of a psychotic illness, known as the “critical period,” is the most crucial in terms of limiting or even preventing the development of disability, with maximal levels of disability reached within the first 5 years after illness onset [3]. The first onset of psychosis typically occurs between the age of 18 and 24 [4], although prodromal symptoms are often detectable in the adolescent years [5]. There is a paucity of research that has focused investigation on the “functional” outcome of this early psychosis cohort. A systematic review of 37 early detection/intervention studies in first-episode psychosis (FEP) found that 42% of patients had “good” illness outcomes as opposed to 27% who had “poor” outcomes at an average of 3-year follow-up [6]. However, outcome was predominantly defined by hospital readmission or change in symptom severity, with only 4 of the 37 studies reporting functional recovery. Another review, which specifically focused on social, role/occupational, and community functioning, found long-term functional outcome to be poor for a substantial proportion of FEP patients [7]. Furthermore, two randomized controlled trials (RCTs) of intensive therapy, involving multidisciplinary teams offering a mixture of cognitive behavioural therapy (CBT), family therapy, vocational interventions, and social skills training, failed to find significant changes in the size of patients' social network [8, 9] or the amount of contact with existing friends [8]. In addition, although the latter study found more patients in the intensive therapy group (49%) to have spent at least 6 months in educational/vocational activities, a large percentage of patients who were considerably impaired in their role functioning at the 18-month follow-up remained. Overall, findings support early intervention strategies as key to the treatment and prevention of psychotic disorders, which has shown significant benefits over more traditional approaches [10]. However, this biopsychosocial approach [11] is largely ineffective for the treatment of social impairment, highlighting the need for innovative intervention strategies targeting functional outcome.

This paper will examine the evidence and potential for neurocognitive and social cognitive interventions in addressing the issue of functional recovery in early psychosis. With the impetus that cognitive deficits are at the core of schizophrenia [12], this paper will only include interventions that have a neurocognitive or social cognitive target; thus, interventions that focus primarily on changing behaviour, such as social skills training (SST) and cognitive behavioural therapy (CBT), will not be addressed here (for review of other interventions see [13]). For this review, the term “neurocognition” will be used to refer to elementary cognitive abilities (e.g., memory, attention, etc.) and remediation/therapies that are neurocognitive based. “Social cognition” which encompasses the perception, interpretation, and processing of social information, will refer to purely social cognitive abilities, primarily, emotion perception (recognition of facial and vocal affect); social perception and social knowledge (the ability to detect and comprehend social cues with respect to social context); Theory of Mind (ToM; the mental capacity to infer one's own and others' mental states); attributional style (tendencies in explaining the cause of events, i.e. to the self, others or the environment) [14]. The term “cognition” will be used to encompass both neurocognition and social cognition. We will first provide a rationale for focusing on cognition by drawing on evidence supporting the neurodevelopmental hypothesis and the link between cognition and functional outcome. This paper will then highlight the differences in the theoretical underpinnings of the available interventions, in addition to reviewing original research that has tested the effects of such interventions on functional outcome in FEP or ultra high-risk (UHR) of psychosis samples. Discussion surrounding future pathways for achieving real-world improvements in social and occupational functioning will be provided, within the context of the early phase of illness.

## 2. Why Focus on Cognitive-Based Interventions?

### 2.1. The Neurodevelopmental Hypothesis and Evidence from Neuroimaging Research

While basic cognitive skills are acquired by early childhood, research suggests higher-order cognitive abilities are refined during adolescence [15, 16]. This coincides with substantial changes in white and grey-matter volume as part of normal neurodevelopment [17]. Regions that appear to undergo substantial structural and functional changes are those particularly associated with response inhibition, evaluation of risk and reward, and emotion regulation [18], domains falling under the broader rubric of executive neurocognitive and social cognitive abilities. Notably, changes are occurring at a time when there is a corresponding shift in social behavior, given adolescence is typically a tumultuous period of life where an individual becomes more self-conscious, heavily influenced by peers, and begins to develop a personal identity [19]. Such brain and behavioural changes, along with pubertal hormonal changes, are thought to be causal factors for the higher rate of psychiatric illness onset observed during adolescence [20]. For example, in Australia, individuals aged between 14 and 25 have the highest prevalence (26%) of a current diagnosed mental disorder [21]. Approximately 65% of Australians who develop a psychotic disorder experience their first episode before the age of 25, with 23% experiencing psychosis onset between age of 25 and 34 [22]. Worth noting, almost 40% first experience psychotic symptoms in their teenage years. Taken together, the abnormal maturation of specific brain regions during this critical period may be related to cognitive deficits in schizophrenia and early psychosis. Furthermore, the chances of cognitive training/therapy being absorbed and retained is likely to be higher due to the greater brain plasticity associated with ongoing neurodevelopment in this adolescent/early-adult phase of life [23].

In FEP, impairments are present in all domains of neurocognition, with the most severe deficits observed in immediate verbal memory and speed of information processing [24, 25]. The profile and severity of neurocognitive impairment tends to be stable over time and is similar to that seen in chronic schizophrenia [26]. Findings regarding the UHR cohort are less consistent [27], but similarly tend to suggest general impairment with accentuated deficits specifically in verbal memory, working memory, and processing speed for individuals who later convert to full-threshold psychosis [28]. A recent meta-analysis of functional magnetic resonance imaging (fMRI) data has shown that both FEP and UHR patients display neurocognitive abnormalities most commonly related to the prefrontal and anterior cingulate cortices, the basal ganglia, hippocampus and cerebellum [29]. In FEP specifically, abnormal blood-oxygen-level-dependent (BOLD) responses were observed in the regions of interest (dorsolateral and ventrolateral prefrontal cortices and anterior prefrontal cortex) during tasks assessing working memory, verbal fluency, executive control, elementary information processing, context processing, and planning/logical reasoning, with medium to large effect sizes (ESs).

The study of social cognition in early psychosis is less advanced. Despite this, compelling evidence indicates that multiple social cognitive domains are dysfunctional in FEP, including emotion recognition [30–34], social perception [34–36], ToM [34, 37–39], and attributional style [40, 41]. Research into social cognition in UHR is scarce; however, review of the limited literature (see [42]) suggests deficits are present in emotion recognition [33, 34, 43–45], ToM [34, 46], and social perception/knowledge [34, 47], as well as bias in attributional style [41]. A recent cross-sectional study involving three patient groups at different developmental stages of the illness (i.e., prodromal, FEP, and chronic) provides further evidence for social cognitive deficits presenting early during the at-risk phase, which remain stable through the later illness phases [34]. Brain regions that are typically activated during social cognitive tasks include the medial prefrontal cortex (mPFC), orbitofrontal cortex (OFC), superior temporal sulcus (STS), temporoparietal junction (TPJ), cingulate cortex (CC), amygdala and anterior insula, collectively known as the “social brain” [48]. Numerous imaging studies have found these regions to be structurally abnormal in FEP and UHR (for review see [49, 50]). To date, only one study has examined brain activation during a social cognitive (emotion discrimination) task in adolescents with early-onset schizophrenia [51]. Although performance on the task was unimpaired, patients showed decreased activation in areas such as the CC and insula during facial emotion processing. Two studies in UHR patients have also found abnormal activation of social brain regions in relation to emotion processing [52] and ToM mental state attributions [53].

Evidence of abnormal anatomical and functional connectivity between frontal and temporal/parietal regions in early psychosis [54–57] further implicates dysfunctional brain networks that govern cognitive processes as central features in illness pathophysiology. Overall, neuroimaging evidence highlights cognitive deficits as being at the core of psychotic illness and supports general cognition, plus several specific domains, as good candidates for targeted interventions during the early phase of illness.

### 2.2. The Role of Neurocognition and Social Cognition in Functional Outcome

For well over a decade neurocognition has been acknowledged as having a significant impact on functioning and functional outcome [58]. We will use the term “functional outcome” in relation to prospective studies and follow-up trials, while the term “functioning” will generally refer to the current level of functioning at the time of testing. Functional outcome/functioning are usually measured by assessment of actual performance of activities of daily living/social activities (e.g., hours worked) or assessment of the ability to perform everyday functioning skills (e.g., using a role-play task). Systematic reviews in chronic schizophrenia suggest that cognitive deficits (especially in executive functioning, verbal learning, and memory and attention) have an equal or greater role than psychotic symptoms in predicting current and prospective functional impairment [58, 59]. Results are less consistent in FEP, most likely due to methodological issues; however, findings currently lean towards verbal/language skills most consistently predicting functional outcome [60].

While debate continues over the degree of overlap between neurocognitive (in particular attention/executive control, basic reasoning) and social cognitive processes, research supports these two domains as independent constructs that contribute unique variance to the prediction of functional outcome, social functioning, and interpersonal skills in both chronic schizophrenia and early psychosis [30, 61–65]. Social cognition, in addition to conferring direct effects, has been suggested to act as a mediator between neurocognition and social functioning [30, 66, 67]. Worth noting, social cognition has been argued to have a greater impact on work-related social skills and work outcomes than neurocognition [61, 68, 69]. A recent meta-analysis concluded that social cognition was a stronger predictor of overall community functioning, incorporating independent living skills plus social and work functioning, than general neurocognition [59]. The few studies that have examined the relationship between social cognition and functioning in FEP support the findings of chronic schizophrenia research. Specifically, emotion recognition [30, 66], emotional intelligence/social perception [65, 66, 70], and social knowledge [70] have all been shown to have direct or indirect effects on general social functioning, and in some cases interpersonal problem solving as well. Findings from a recent 12-month follow-up study further highlight the significant longitudinal impact of social cognitive deficits on work, independent living, and social functioning outcomes [71].

Despite some discrepancies in the literature and the limited studies in FEP, evidence from chronic schizophrenia research is robust in that cognition undeniably has a significant impact on functional outcome. In terms of neurocognition and social cognition, verbal fluency and ToM respectively, are shown to have the largest effects on community functioning [59]. Further research is needed to determine the impact of these two cognitive domains on alternative areas of functioning and functional outcome, such as social behaviour, social problem solving, and social skills.

Overall, behavioural findings coupled with neuroimaging evidence suggest that there is potential for significant improvement and even prevention of social and occupational decline for young people with emerging and first-onset psychosis and that interventions targeting cognitive processes are warranted.

## 3. Background of Cognitive Intervention Programs in Schizophrenia

Cognitive remediation is the most common form of the cognitive-based interventions used in schizophrenia and has recently been defined as “a behavioural training based intervention that aims to improve cognitive processes (attention, memory, executive function, social cognition or metacognition) with the goal of durability and generalization” [72, page 1]. This definition implies that although dysfunction in cognition, including social cognition, is the intervention target, improved and sustained functioning in the real world is the desired outcome [73]. It should be noted that, although social cognition is included in the above definition, the majority of cognitive remediation programs to date have predominantly targeted neurocognitive processes. The approaches used in cognitive remediation are highly variable, but a clear common element is repeated practice in exercises requiring effortful “thinking skills.” The majority of programs also involve a trained facilitator (see [74] for exception). However, the frequency and duration of training sessions is inconsistent, as is whether the intervention is delivered one-on-one or involves a group-based component.

Numerous systematic reviews of neurocognitive remediation have been published, but the first quantitative meta-analysis (26 studies) to examine the effects of neurocognitive remediation on functional outcome found a small-to-medium mean ES of 0.36, which was only slightly lower than the improvement found in neurocognitive function (mean ES 0.41) [75]. Interestingly, the effect of neurocognitive remediation on functioning was significantly moderated by whether or not adjunctive psychosocial rehabilitation, such as vocational programs or SST, was provided, with the finding that provision of psychosocial rehabilitation led to significant improvements in social functioning (mean ES 0.47). In comparison, studies of neurocognitive remediation alone produced a meagre mean ES of 0.05. These findings were broadly replicated in an updated meta-analysis that included 40 RCTs, although the effect sizes were slightly larger [72]. The mechanism for this “synergistic” effect is hypothesised to be the improved neurocognitive functioning produced by neurocognitive remediation enhancing the capacity to learn new skills, which in turn leads to increased benefit from psychiatric rehabilitation [75]. However, this hypothesis requires further empirical investigation, such as examination of whether change in cognition is associated with change in functioning.

Because social cognitive neuropsychiatric research is in its infancy, social cognitive interventions are considered to be a relatively new line of treatment for improving social functioning. The primary goal of such interventions is to enhance social cognitive abilities, which as mentioned earlier, are considered to be largely independent constructs to neurocognitive processes. Social cognitive interventions typically employ a model that is bidirectional and flexible, where social cognitive processes, emotions, and actions are addressed in unison or in parallel, as opposed to a pure bottom-up information-processing framework characteristic of some neurocognitive interventions. Due to the limited research into social cognitive interventions, fewer reviews have been conducted. The most commanding review to date is a recent meta-analysis of 19 controlled trials of social cognitive training in established schizophrenia [76]. A large mean ES (0.78) was found for observer-rated indices of functional outcome (6 of the 19 studies), which in contrast to the neurocognition meta-analyses was larger than most of the ESs found for specific domains of social cognition. This finding is impressive given that the social cognitive interventions reviewed led to generalised improvements in functioning mostly in the absence of adjunctive psychosocial interventions. It has been suggested that the mediating role of social cognition between neurocognition and functional outcome implies that social cognition is more proximal to real-world functioning. Thus, targeting social cognition may produce greater effects on functioning than neurocognition [77].

Given that this paper is focused specifically on early psychosis, it is important to explore whether age or stage of illness may be a key moderator of outcome in trials of cognitive remediation. With respect to participant age as a moderator, the existing research is equivocal. The McGurk et al. [75] meta-analysis found that older patients (aged 38–50 years) benefited more from neurocognitive remediation in terms of functioning (mean ES 0.55) than younger patients (aged 15–37 years; mean ES 0.18). However, it should be noted that the younger patient group included a wide age range and likely included patients with a long duration of illness. The more recent meta-analysis by Wykes et al. [72] found that age did not moderate outcome, but it was unclear as to whether this included functional outcome. Conversely, the Kurtz and Richardson [76] meta-analysis found that age was negatively correlated with functional (and symptomatic) outcome, such that younger patients (youngest mean age was 25 years) benefited more from social cognition training than older patients, although exact age ranges were not stated. Some studies have suggested that illness stage (e.g., first-versus multiple-episode) or duration of illness affects responses to cognitive remediation, but again findings are mixed. Longer illness duration is associated with poorer response to neurocognitive remediation [78–82], but better response to social cognitive remediation [76]. Despite the majority of studies to date having recruited patients with longstanding psychotic disorders, evidence from neuroimaging and behavioural research discussed earlier suggests that intervening in the early stages of psychosis may provide greater potential for recovery to premorbid levels, when individuals are less removed from their original functional trajectories.

## 4. Current Approaches for Treating Cognitive Deficits

Having briefly reviewed the evidence for cognitive remediation in schizophrenia, the paper will now turn to describing the specific approaches used and their theoretical underpinnings.

### 4.1. Drill and Practice

Also referred to as the stimulation approach [83], drill and practice is the most common technique used in neurocognitive remediation programs for psychosis [72, 75]. Restoration or enhancement of cognitive function is the goal of drill and practice, and the underlying model presupposes neural plasticity and that cognitive impairment can be rectified, subsequently leading to improvements in performance in daily activities. In drill and practice, individuals are engaged in focused cognitive processing by repetitive training on progressively more difficult exercises. Usually these exercises are computerized, but may also be paper and pencil, and can be theoretically categorized as either “bottom-up” or “top-down.” Bottom-up approaches presume that a basic deficit in signal-to-noise discrimination of auditory and visual information underlies higher-level cognitive dysfunction. An example of a bottom-up drill and practice approach is Targeted Cognitive Training (TCT) using the PositScience program [74, 84], which usually involves daily sessions totaling 50 to 100 hours [74, 84]. Top-down drill and practice approaches, such as the Neuropsychological Educational Approach to Cognitive Remediation (NEAR) [85], target higher-order cognitive functions (executive, complex attention, memory) usually via exercises that simultaneously harness a broad range of cognitive abilities, believed to simulate one's cognitive engagement in real-world activities [73].

### 4.2. Strategy Coaching

Strategy coaching is a technique commonly used in cognitive remediation programs, usually in conjunction with drill and practice [72]. However, it is not well defined. There is some conjecture as to whether this approach involves transfer of remediated cognitive abilities to daily functioning (i.e., generalised restoration) or assists individuals to compensate for their cognitive deficits conferring improved functioning [75, 86]. Strategy coaching includes teaching individuals to use specific mental strategies to enhance performance on specific exercises or tasks, for instance, teaching a person to use visualisation/mental pictures to help them remember a list of words. An example of a program that relies heavily on strategy coaching (in addition to drill and practice) is Cognitive Remediation Therapy (CRT), adapted by Wykes and Reeder [87] from the work of Delahunty and Morice [88]. CRT is an individualised one-on-one 40-hour (3 times/week) teaching program aimed at enhancing working memory, planning, and problem solving (cognitive flexibility), with the goal of transferring cognitive skills to real-world functioning.

### 4.3. Compensation

Alternative approaches which are not traditionally used in cognitive remediation [72], but are used in other neurocognitive interventions, include compensation and adaptation [89–92]. The model underlying compensatory approaches assumes that developing alternative strategies will reduce cognitive load and minimize the impact of cognitive impairment on daily functioning, in much the same way as glasses reduce the impact of vision impairment [93]. Compensatory approaches can be broadly categorised into three techniques: self-management, environmental adaptation, and errorless learning.

*Self-management techniques* include internal (mental) and external (behavioural) strategies. An example of a self-management compensatory program is Cognitive Training (CT) [89]. CT is a manualised 12-week group-based intervention (although it can also be delivered individually) that focuses on four key cognitive domains: (1) prospective memory—remembering to remember (e.g., daily calendar use), (2) attention and vigilance (e.g., using self-talk to remain on task), (3) learning and memory (e.g., mnemonic strategies such as chunking or rhyming), and (4) executive functioning—problem solving and cognitive flexibility (e.g., six-step problem solving technique). Each 2-hour session includes didactic teaching, demonstration/observation, practice, and feedback as well as homework for further practice in daily life. The expectation is that the generic strategies taught in CT will be independently applied by participants to manage their individual day-to-day tasks [89, 94].

The second (not mutually exclusive) approach is *environmental adaptation*, which is applied by an individual and/or caregiver for the purpose of bypassing cognitive deficits. Environmental adaptation techniques generally aim to direct or capture a person's attention so as to prompt performance in goal-directed behaviour at the appropriate time. An exemplar of this approach is Cognitive Adaptation Training (CAT [91]). CAT is a manualised treatment involving the application of multiple external compensatory strategies (e.g., reducing distracting information at a work desk) and environmental supports (e.g., signs, alarms, checklists) that are individually tailored based on initial assessment of level of cognitive dysfunction, behavioural/learning type, and specific functional goals. A broad range of functional domains are targeted, ranging from grooming/personal care to leisure activities, and medication adherence. CAT is delivered in the community (usually at home) on a weekly basis for 9 months [95].

The final compensatory approach that warrants mention is *errorless learning*, which is a strategy used to compensate for a learning deficit in which there is difficulty distinguishing between correct performances and mistakes, even after feedback (which has been shown in schizophrenia [96–98]). Typically, a trainer prevents the individual from experiencing errors while learning a specific task, so that only correct responses are allowed [96]. The task in question (such as learning someone's name or performing a specific task at work) is learnt with a trainer via repetitive practice of smaller simpler task components, gradually working towards total task completion in a stepwise fashion without making any errors. Errorless learning approaches have been most extensively examined by Kern and colleagues with a strong focus on functional outcomes [96, 99, 100]. Like CAT, errorless learning is applied in a highly specific and individualised manner. This may be viewed as either a strength or limitation, depending on the desired outcome of the patient and the researcher/clinician.

### 4.4. Behavioural Learning

Behavioural learning approaches also form an important part of many cognitive rehabilitation interventions. One of the theories underlying behavioural learning is that motivation may impede performance in cognitive or functional tasks in schizophrenia [73, 83, 95, 101, 102]. These approaches are not usually used in isolation and are inherent to many cognitive rehabilitation programs, although often programs do not explicitly state this when describing their protocol. The simplest example is positive reinforcement via praise and encouragement delivered by cognitive specialists or therapists. Some programs explicitly target motivation to complete cognitive exercises via extrinsic monetary incentives [74, 103], while others target intrinsic motivation by using engaging and enjoyable tasks, allowing participant control over learning, and linking these tasks with individual goals [85, 104]. Other examples of behavioural learning approaches include setting tasks at the appropriate level to ensure the participant experiences success [74, 103, 104], shaping—rewarding successive approximations of the target goal [105], and scaffolding—instructional teaching and support in learning something new by building upon what is already known [106].

### 4.5. Metacognitive Learning

Metacognitive rehabilitation strategies, traditionally suggested as an adjunct to CBT for reducing negative beliefs about failure, stigmatization, and coping abilities, have more recently been integrated into cognitive intervention programs [107]. Metacognition can be defined as “thinking about one's thinking” [108], and more specifically knowing about one's own cognitive abilities, states of knowledge, and actual performance, which is distinct from the cognitive abilities, states of knowledge, and performance themselves [109]. Metacognition involves self-reflection, the ability to monitor decision-making and competence, and report-control (i.e., directing behaviour based on thought monitoring) [109]. Metacognitive strategies are almost always coupled with mediated learning and exposure to new experiences, and are therefore frequently incorporated with strategy coaching. For example, Metacognitive Skills Training (MCT) [108] is designed to facilitate participants' awareness of cognitive difficulties, to critically reflect on these difficulties, and to complement or change their current thinking styles accordingly. MCT addresses impairments in both neurocognitive and social cognitive domains by focusing on issues of self-serving bias, jumping to conclusions, bias against disconfirmatory evidence, deficits in ToM, overconfidence in memory errors, and depressive cognitive patterns.

### 4.6. Role-Play

Role-play, the acting out/simulation of one or more characters in a given social situation, has long been used in SST and as a way for assessing whether the desired behavioural skills were retained [110]. Within a cognitive-based model, role-play is used as a slightly different training tool in two particular ways. One is where cognitive skills are first taught (e.g., using Socratic questioning and metacognitive strategies), and participants are later asked to role-play a social scenario using the taught cognitive skills as practice for real-world application. Role-play is also helpful for the facilitator to determine what areas require further work and tailor the remaining training sessions accordingly. Secondly, similar to modeling, for interventions that involve more than one facilitator (such as Social Cognition and Interaction Training (SCIT) [111]), role-play may be conducted by the facilitators themselves and used as a demonstration tool. This, along with mimicry, is primarily used in social cognitive interventions, as it is thought to engage the simulation system of the brain [112], which has been linked to ToM, emotion perception, and empathic ability [113].

### 4.7. Exposure to Social Situations and Group-Based Therapy

Exposure therapy is fundamentally linked to behavioural learning approaches and is the most established psychosocial intervention for the treatment of social anxiety disorders [114]. Exposure therapy is based on the theory of experimental “extinction,” which is the progressive weakening of a habit via the repeated evocation of the response without reinforcement [115, 116]. Use of exposure traditionally begins with identifying a hierarchy of the feared social situations, which the person then endures and works through starting with the least distressing situation to the point where the anxiety has decreased, before moving on to the next least anxiety-provoking situation [117]. This exposure therapy technique has predominantly been adapted for social cognitive interventions, where the technique is used in a less explicit way by means of group-based therapy and through administration of cognitive-based exercises requiring partner work. The design of SCIT, for example, integrates this technique in a subtle way by assuming social situations are inherently daunting for patients. As such, the level of participation required, and personal relevance to each individual, increases from week to week as group alliance grows [118]. The exposure technique, coupled with role-play, has also been used in Cognitive Enhancement Therapy (CET) [119], where patients are asked to give a 5–10 minute presentation on facts about themselves, qualities in themselves that they admire, examples of these qualities, and why they admire them. In addition, the participants take turns acting as “chairperson” during group discussions.

### 4.8. Theoretical Considerations

Many of the approaches and techniques described previously, particularly those used in social cognitive interventions, feature centrally in traditional CBT and SST. Although theoretically they differ in terms of their treatment target (cognition versus behaviour), it begs the question: how do cognitive interventions differ practically to CBT and SST? The main difference lies in the component of the technique that is emphasized or focused on. For example, in CBT clinicians will typically challenge the *logic* behind specific overvalued beliefs, while neurocognitive interventions conversely focus on the perceptual and interpretive *processes* that may have contributed to the development/maintenance of the delusional belief. SST on the other hand may, for example, use behavioural learning or role-play techniques to reinforce a certain socially appropriate behaviour. However, the underlying thought processes that relate to recognizing and interpreting when a social situation calls for a given emotional and subsequent behavioural response are not emphasized in SST, as they are in social cognitive programs. Lastly, the way in which particular approach strategies are combined will additionally distinguish cognitive interventions as unique from SST and CBT.

## 5. Effects of Neurocognitive Interventions on Functional Outcome in Early Psychosis

### 5.1. Ultra High-Risk

To the best of our knowledge there is only one published study of neurocognitive remediation in individuals at UHR for psychosis [79], with at least one other trial underway [120]. Functional outcome was not measured in the Rauchensteiner study, but given the novel application of cognitive remediation in this population, we will describe it briefly. Rauchensteiner et al. [79] conducted a small pilot study comparing the effects of 10 sessions of primarily drill and practice computer-based cognitive remediation (using Cogpack software) in UHR participants versus a schizophrenia control group. Only the UHR participants improved in cognitive performance following 4 weeks of training, specifically on tests of attention and verbal memory. Notwithstanding significant limitations, including the small sample size, possibility of practice effects, no measure of functioning, convenience sampling, and lack of a control condition, this study provides the first evidence of the potential advantages of delivering cognitive remediation at the putative earliest stage of schizophrenia. Given that cognitive deficits are significantly associated with functional outcome in ultra high-risk individuals [121, 122], there is clear rationale for further research into cognitive remediation in this population.

### 5.2. Recent-Onset Psychosis

Three research groups have published results on different cognitive remediation interventions in recent-onset psychosis and one other is underway [120]. Each of the completed trials will be described with a specific focus on functional outcome. In a small RCT, Ueland and Rund [123, 124] investigated whether 30 hours of individual neurocognitive remediation could enhance functional outcomes in inpatient adolescents (aged 12–18) with early-onset psychosis already receiving comprehensive psychoeducational treatment. Participants were randomized to receive cognitive remediation (*n* = 14) or treatment as usual (TAU; *n* = 12). Cognitive remediation comprised four modules: cognitive differentiation (basic conceptual cognitive skills), attention (enhanced instructions and monetary reinforcement), memory (practice and strategies on verbal and visual memory tasks), and social perception (training in encoding social stimuli). This program involved drill and practice, strategy coaching, and behavioural learning techniques. Both groups improved in cognition and functioning over time, with no significant between-group differences found in functional outcome (as measured by the Global Assessment Scale) at post-intervention or 1-year follow-up. Furthermore, changes in cognition were not associated with changes in functioning.

The second study was an RCT of CRT (described previously) for patients with recently diagnosed adolescent-onset schizophrenia (aged 14–22) [125]. Participants were randomly allocated to CRT (*n* = 21) or TAU (*n* = 19). Despite CRT effecting improvement in cognitive flexibility relative to the control condition, there were no group differences in functioning as measured by the Social Behaviour Schedule. However, secondary analyses found that improvement in cognition was associated with improvements in social functioning in the context of CRT, providing preliminary evidence for restitution of cognitive function being associated with functional improvements.

Finally, the largest and longest RCT of cognitive remediation in recent-onset psychosis (defined as onset < 8 years prior) was conducted by Eack and colleagues [126]. Participants were randomized to receive CET (*n* = 31) or an active control condition: Enriched Supportive Therapy (EST; *n* = 27). CET [119] is a comprehensive program that integrates remediation in neurocognition and social cognition using multiple methods including 60 hours of computer-assisted exercises (attention, memory, and problem solving; PSSCogRehab program) conducted in pairs. It also includes 45 group-based weekly training sessions in social cognitive skills (perspective taking, social perception, managing emotions, and social context appraisal) taught and practiced *in vivo* via psychoeducation, role-play, and experimental and homework exercises. The program takes 2 years to complete. CET is unique in that it is the only cognitive remediation approach that comprehensively addresses both neurocognition and social cognition. EST is an individual therapy that focuses on psychoeducation and stress management, but is not matched to CET in session number or program duration. At 2 years (post-intervention), relative to EST, CET resulted in significantly greater improvements in almost all composite domains of neurocognitive and social cognitive function, as well as a composite index of social adjustment (Social Adjustment Scale-II, Major Role Adjustment Inventory, Global Assessment Scale, and Performance Potential Inventory) [126]. Further analyses revealed that CET participants achieved superior outcomes in terms of competitive employment (58% versus 19%), wages earned, work readiness, employment satisfaction, social relationships, social functioning, social leisure, role adjustment, activities of daily living, and instrumental task performance, as well as global functioning [126, 127]. These functional gains were largely maintained at 1-year follow-up [128]. One major caveat to these findings is that raters were not blind to treatment allocation.

An important question arising from this study is what are the active ingredients of CET catalysing the functional improvements observed? The largest effects were found in the domain of social cognition with over 60% improvement over 2 years compared to just over 10% improvement in neurocognition over the same period [126]. This possibly implicates the social cognitive training aspect of CET as the more active ingredient. Interestingly Eack et al. [128] found that improvement in neurocognition by treatment end was the only predictor of maintenance in social adjustment at 1-year follow-up. Subsequent analyses of specific employment outcomes found that improvements in both social cognition and neurocognition mediated the effects of CET on employment [127]. Notwithstanding these impressive findings, a second major caveat to this study is that the CET group received many more hours of therapist contact and socialization in pairs/groups compared to the control group, so it is possible that greater exposure to social situations and therapist support/guidance resulted in the improved functional outcomes. The issue of feasibility should also be questioned and whether CET can be supported within a clinical service given the additive level of input required by the treating clinical team and the sheer amount of time involved. It is also worth noting that, at baseline, participants had a mean age of 25.9 years (SD = 6.54 years) and a mean illness duration of 3.2 years (SD = 2.2) [126], with 22% of individuals having been unwell for more than 5 years (<8 years). Thus, findings may not be generalised to conventional definitions of early psychosis (which is typically within 2 years of first illness presentation). Further research is needed to tease out the active elements in CET as well as other remediation programs that are associated with improvements in functional outcome [73, 83, 129].

## 6. Effects of Social Cognitive Interventions on Functional Outcome in Early Psychosis

Due to the more recent inception of social cognition in the field of schizophrenia research, no published literature to date has investigated a social cognitive intervention program in a UHR or FEP cohort. Despite this, the meta-analysis of chronic schizophrenia studies mentioned earlier provides very promising findings [76]. We will briefly review the available interventions that have been trialed in established schizophrenia, with reference to their impact on functional outcome and applicability in early psychosis.

Social Cognition and Interaction Training (SCIT), developed by Penn et al. [130], is probably the most evidence-based stand-alone social cognitive intervention currently available. SCIT is a manualised 20-week (1 session/week) group-based intervention targeting multiple aspects of social cognition. SCIT employs a range of approaches including behavioural learning (and at times strategy coaching), metacognitive training, role-play, and exposure therapy techniques. There are three phases: (1) emotion training, which focuses on defining emotions, emotion mimicry, and understanding paranoia, (2) figuring out situations, which focuses on distinguishing facts from guesses, jumping to conclusions, and understanding bad events, and (3) integration, which encourages participants to “check it out” or apply their newly acquired social cognitive skills to personal real-life situations. A small trial that compared SCIT (*n* = 18) to a coping skills group (*n* = 10; which focused on problem-solving, symptom management, and relapse prevention) found SCIT to be significantly more effective at improving performance on measures of social and emotion perception, ToM, and attributional style [111]. Aggressive behaviour, patient's need for closure, and social relationships also improved with SCIT. Moreover, social functioning (as assessed with the Social Functioning Scales: engagement and interpersonal [131]) improved significantly, independent of changes in psychopathology. A more recent trial of SCIT in an outpatient setting has yielded similar positive results [132]. These successful trials led to the integration of the SCIT program into standard clinical practice within mental health services around New York [133]. Given the SCIT model emphasizes that performance varies as a function of the personal relevance and emotional tone of the social context and ultimately aims to improve day-to-day living, our research team has adapted SCIT to be suitable for young people with emerging psychosis. A pilot study in a FEP sample is currently underway.

Emotion and ToM Imitation Training (ETIT) [134] is one of the other few social cognition interventions that have been found to enhance functional outcome. This is a 12-week group-based program, involving participants imitating emotional facial expressions in the mirror, tracking eye-gaze of others, and observing pictures of art, sketches, and comic strips depicting social scenarios with the aim of enhancing the ability to infer mental states of others and attribution of intentions. ETIT stems from the mirror neuron theory of imitation [135], thus relies heavily on the mimicry approach, but also uses coaching and role-play techniques. A small RCT found that schizophrenia patients in the ETIT group (*n* = 16) improved significantly on multiple aspects of social cognition, but also on a global measure of personal and social functioning, in comparison to individuals in a problem solving skills training group (*n* = 17) [134]. To our knowledge no follow-up studies or trials in an early psychosis population have been published.

There are several other targeted and broad-based social cognitive interventions that have been developed and trialed in schizophrenia, but to date most have conducted limited assessment of functional outcome. However, as found by Kurtz and Richardson [76], significant effects on functioning are emerging. One of the programs available for possible use in FEP samples is Social Cognition Enhancement Training (SCET) developed by Kwon [136] and modified by Choi and Kwon [137]. This is a 6-month (36 session) group-based program consisting of three phases (elementary, middle, and advanced), each with 12 sessions. The use of cartoon stories is a central feature of SCET, which aid in the learning of social cue perception, context appraisal, and social knowledge. Additional techniques used are psychoeducation, behavioural learning approaches, and exposure to social situations via group work. Other programs include MCT (described earlier) and a similar intervention devised by Roncone et al. [138], Training of Affect Recognition (TAR) [139], and Ekman's Micro-Expression Training Tool (METT) [140], but comprehensive studies are lacking, even in established schizophrenia [76]. Nevertheless, these interventions and various techniques may still hold potential for enhancing social and other areas of functioning. However, larger studies that include functioning as a primary outcome across different illness stages are required before any inferences can be made.

## 7. Conclusions and Future Directions

The literature is clearly lacking in the area of early psychosis, with only four studies having trialed a cognitive intervention, and these were primarily focused on neurocognition as opposed social cognition. With respect to functional outcome, the most successful of these appeared to be CET. However, as discussed, the CET trial had significant limitations. These included the control group being unmatched for nonspecific treatment effects and raters not being blind to group allocation, making it difficult to draw firm conclusions regarding the potency of specific aspects of the intervention (i.e., neurocognitive versus social cognitive remediation). Furthermore, participants were not representative of traditional early psychosis samples.

Our review revealed that there are currently no published data available on the efficacy of an exclusive social cognitive intervention in an early psychosis sample. This is noteworthy, given that the behavioural evidence, discussed earlier, and the quantitative review revealing larger effects of social cognitive training on functioning relative to reviews of cognitive remediation in established schizophrenia [76], points towards social cognition as having a greater impact on functional outcome than neurocognition.

In summary, it is presently impossible to make any definitive conclusions regarding the usefulness of cognitive-based interventions in the treatment of functional disability for this early illness population.

While there is evidence that cognitive interventions lead to significant moderate improvements in specific cognitive abilities for individuals with established schizophrenia, residual cognitive deficits often remain [72, 75]. It has been previously suggested that there may be a ceiling to the effects of cognitive remediation at least when delivered in isolation [92]. At present, it is unclear how much improvement in cognition is enough to lead to significant functional improvements, whether stand-alone cognitive interventions are sufficient, or even whether improvement in cognition is essential for functional recovery. Furthermore, it is entirely possible that psychosocial programs that only target functioning may improve cognition, in the absence of a program specifically aimed at treating cognitive deficits [141]. These points open a number of important avenues for future research, with the overarching question being how do we optimize functional outcome?

Starting with evidence from neurocognitive interventions, the schizophrenia literature indicates that neurocognitive remediation combined with psychosocial rehabilitation is far superior with respect to functional outcome than when cognitive remediation is delivered alone [72, 75], yet apart from the Ueland and Rund [124] study, no other trials combining psychosocial with cognitive approaches have been completed in early psychosis, although at least two others are currently underway [142, 143]. One RCT proposes to combine Multifamily Group Psychoeducation with neurocognitive remediation using the PSSCogRehab computer program (drill and practice approach), with the primary outcome being relapse rates. Secondary outcomes include functioning in social, vocational, and activities of daily living domains and quality of life in patients with recent-onset psychosis [143]. Another RCT (NEUROCOM study) will recruit FEP patients and randomize them to receive a combination of cognitive training with a comprehensive psychosocial program (OPUS) or OPUS alone, with the primary outcome being everyday functional capacity [142]. In this study the cognitive training will comprise a combination of computer-assisted drill-and-practice exercises and strategy coaching as well as teaching compensatory strategies (e.g., calendar use). The outcomes of these studies will be very informative.

Despite the lack of early psychosis research into the combined intervention approach, Integrated Psychological Therapy (IPT) [144] has been extensively investigated in established schizophrenia and warrants discussion. IPT is a manualised group-based intervention that aims to ameliorate cognitive impairment and social behavioural deficits, with the ultimate goal of improving social competence [145]. It involves 30–60 min sessions three times per week for 3 months and comprises 5 hierarchically arranged subprograms targeting cognitive differentiation, social perception, verbal communication, social skills, and interpersonal problem solving. Meta-analysis of 30 IPT studies (7 of which were RCTs) showed that IPT compared to a control condition or TAU was more effective for enhancing neurocognition (mean ES 0.41), psychosocial functioning (mean ES 0.31), and psychopathology (mean 0.31) [146]. The largest effect was on neurocognition, which is not surprising given IPT is heavily focused on this domain. Not only do the findings support the combined approach as putatively superior to stand-alone interventions, but further implicate the importance of early intervention, given that duration of illness was the only negative predictor on the global therapy outcome of IPT.

One question that cannot be answered from these combination studies is whether targeting neurocognition or social cognition will be more fruitful regarding functional outcome, and furthermore, which approach strategies/techniques or combination of techniques are likely to have the biggest impact on functioning. We suggest that social cognitive interventions, which typically rely on approaches such as metacognitive learning, didactic teaching and exposure techniques via group-based therapy, hold promise for producing robust effects on functional outcome, but to reiterate they are yet to be trialed in early psychosis. Furthermore, while some neurocognitive approaches have been trialed in early psychosis, others have not. For example, evidence suggests that compensatory approaches may play an important role in maximizing functional recovery, and indeed, large effects (>0.80) have been found in individual studies of chronic schizophrenia populations [89, 91, 147]. Head-to-head studies of the various cognitive interventions are required to tease out the most effective techniques.

A recent elegant study by Horan and colleagues [77] attempted to address this. After trialing a modified version of SCIT [148], Horan et al. developed the Social Cognitive Skills Training (SCST) program comprising 24 sessions. This program is similar to SCIT in that it targets emotional processing, social perception, attributional bias and mentalising skills with the primary aim of enabling participants to become more proficient in social interacting. Schizophrenia outpatients were randomized to receive either SCST (*n* = 16), computerized neurocognitive remediation (*n* = 19; exercises developed by PositScience), standard skills training (*n* = 19), or a hybrid of SCST and neurocognitive remediation (*n* = 14) [77]. Groups were matched for session number and time with the therapist. This was the first study to directly examine the effects of combining a social cognitive with a neurocognitive intervention and is also the first study to conduct a well-controlled head-to-head comparison of neurocognitive versus social cognitive remediation. The SCST was more successful in treating deficits in facial affect recognition, when compared to the neurocognitive remediation and standard skills training groups, as well as emotion management, when compared to the neurocognitive remediation and the hybrid groups. The hybrid group, in comparison to the three other groups, improved significantly on the measure of attributional bias. For other social cognitive and neurocognitive measures there were general improvements, but these did not differ between treatment groups. There were no significant improvements on the measures of functional outcome in any group, although a positive trend was observed for both the SCST and the neurocognitive remediation groups on a measure of social competence. The findings suggest SCST as a stand-alone treatment can enhance emotion processing, but functioning was unaffected. However, group numbers were small, and authors suggested that integration sessions conducted within the community may lead to better skill retention and generalisation, but this requires further investigation. It should also be noted that the neurocognitive remediation program was a prototype and appeared to use only the drill and practice approach without utilizing complimentary techniques such as strategy coaching. Furthermore, participants were compensated for attending training sessions, making it difficult to determine the true participant acceptability of the different approaches.

While Horan et al. [77] provide evidence to support the SCST as a successful stand-alone intervention for treating emotional processing deficits, the finding that the hybrid intervention had a significant effect on attributional bias also suggests a combined approach may be useful. This line of work warrants further research, especially given that the hybrid training was matched in number of hours to the other interventions, meaning the amount of time spent on social cognitive training was half that of the SCST group and similarly for the neurocognitive component. However, to reiterate, functioning, which is the outcome of primary interest, was not significantly affected by either the SCST alone or the combined approach. The results from the CET trial [126–128], an intervention that encompasses both neurocognitive and social cognitive training, add further weight to the idea that a combined/hybrid approach appears complimentary and may work synergistically to enhance social and occupational functioning.

Finally, coming back to the neurodevelopmental hypothesis and biological basis of schizophrenia, it would be advantageous for future clinical trials to utilise modern brain imaging techniques not only to explore the origins of cognitive impairment but to evaluate the effects of intervention programs on the illness at its source. Indeed, Eack and colleagues [149] found CET to have a neuroprotective effect against grey matter volume reductions and that CET was a significant mediator between grey matter volume changes in the parahippocampus, fusiform gyrus, and amygdala and improved cognitive performance. Normalization of brain activation [150] and serum brain-derived neurotrophic factor [151] have also been demonstrated in chronic schizophrenia following neurocognitive remediation.

In conclusion, given the evidence for debilitating cognitive and functional difficulties occurring at or even before onset of psychosis and the clear relationship between these two constructs, we argue that the maximal benefits of cognitive remediation are likely to occur early in the course of illness, even in the putative prodromal phase. This is based on the premise that the “ceiling” for potential recovery may be higher in early psychosis due to ongoing neurodevelopment in this phase of life. Needless to say there is a great deal more research to be done in this area; however, preliminary findings suggest there are a number of potential avenues that may alleviate functional impairments of young people with psychotic disorders.
